# Glycosylation Profile of Immunoglobulin G Is Cross-Sectionally Associated With Cardiovascular Disease Risk Score and Subclinical Atherosclerosis in Two Independent Cohorts

**DOI:** 10.1161/CIRCRESAHA.117.312174

**Published:** 2018-03-13

**Authors:** Cristina Menni, Ivan Gudelj, Erin Macdonald-Dunlop, Massimo Mangino, Jonas Zierer, Erim Bešić, Peter K. Joshi, Irena Trbojević-Akmačić, Phil J. Chowienczyk, Tim D. Spector, James F. Wilson, Gordan Lauc, Ana M. Valdes

**Affiliations:** 1 From the Department of Twin Research and Genetic Epidemiology (C.M., M.M., J.Z., T.D.S., A.M.V.); 2 Department of Clinical Pharmacology, British Heart Foundation Centre (P.J.C.); 3 King’s College London, United Kingdom; Genos Glycoscience Research Laboratory, Zagreb, Croatia (I.G., I.T.-A., G.L.); 4 Centre for Global Health Research, Usher Institute of Population Health Sciences and Informatics (E.M.-D., P.K.J., J.F.W.); 5 Medical Research Council Human Genetics Unit, Institute of Genetics and Molecular Medicine, Western General Hospital (J.F.W.); 6 University of Edinburgh, Scotland; National Institute for Health Research Biomedical Research Centre at Guy’s and St Thomas’ Foundation Trust, London, UK (M.M.); 7 Institute of Bioinformatics and Systems Biology, Helmholtz Zentrum München, Neuherberg, Germany (J.Z.); 8 Faculty of Pharmacy and Biochemistry, University of Zagreb, Croatia (E.B., G.L.); 9 School of Medicine, Nottingham City Hospital, United Kingdom (A.M.V.); 10 National Institute for Health Research (NIHR) Nottingham Biomedical Research Centre, United Kingdom (A.M.V.).

**Keywords:** atherosclerosis, cardiovascular disease risk, glycosylation, immunoglobulin G, plaque, atherosclerotic

## Abstract

**Rationale:**

One measure of protein glycosylation (GlycA) has been reported to predict higher cardiovascular risk by reflecting inflammatory pathways.

**Objective:**

The main objective of this study is to assess the role of a comprehensive panel of IgG glycosylation traits on traditional risk factors for cardiovascular disease and on presence of subclinical atherosclerosis in addition to GlycA.

**Methods and Results:**

We measured 76 IgG glycosylation traits in 2970 women (age range, 40–79 years) from the TwinsUK cohort and correlated it to their estimated 10-year atherosclerotic cardiovascular disease risk score and their carotid and femoral plaque measured by ultrasound imaging. Eight IgG glycan traits are associated with the 10-year atherosclerotic cardiovascular disease risk score after adjusting for multiple tests and for individual risk factors—5 with increased risk and 3 with decreased risk. These glycans replicated in 967 women from ORCADES cohort (Orkney Complex Disease Study), and 6 of them were also associated in 845 men. A linear combination of IgG glycans and GlycA is also associated with presence of carotid (odds ratio, 1.55; 95% confidence interval, 1.25–1.93; *P*=7.5×10^-5^) and femoral (odds ratio, 1.32; 95% confidence interval, 1.06–1.64; *P*=0.01) plaque in a subset of women with atherosclerosis data after adjustment for traditional risk factors. One specific glycosylation trait, GP18-the percentage of FA2BG2S1 glycan in total IgG glycans, was negatively correlated with very-low-density lipoprotein and triglyceride levels in serum and with presence of carotid plaque (odds ratio, 0.60; 95% confidence interval, 0.50–0.71; *P*=5×10^-4^).

**Conclusions:**

We find molecular pathways linking IgG to arterial lesion formation. Glycosylation traits are independently associated with subclinical atherosclerosis. One specific trait related to the sialylated N-glycan is negatively correlated with cardiovascular disease risk, very-low-density lipoprotein and triglyceride serum levels, and presence of carotid plaque.

## Abstract 2

Supplemental Digital Content is available in the text.

Cardiovascular diseases (CVDs) are the first cause of morbidity and mortality in Western countries.^1^ In addition, the improvement of treatment and the reduction of case fatality are consistently increasing the prevalence of people who are at risk for recurring events or cardiac decompensation.^2^ Many, often co-occurring, risk factors have been identified and account for most of the CVD burden,^3^ and different validated algorithms have been developed to estimate the individual risk of developing specific CVD events.^4–6^

**Editorial, see p 1488**

**Meet the First Author, see p 1480**

The 10-year atherosclerotic cardiovascular disease (ASCVD)^7^ risk score is a sex- and race-specific single multivariable risk assessment tool used to estimate the 10-year CVD risk of an individual that has replaced clinically the Framingham 10-year cardiovascular risk score.^8^ It is based on age, sex, ethnicity, total cholesterol and high-density lipoprotein (HDL) cholesterol, systolic blood pressure, smoking status, use of blood pressure–lowering medications, and the presence of type 2 diabetes mellitus (T2D). Data on subclinical atherosclerosis, presence of atherosclerotic plaques in carotid and femoral arteries, used in combination with traditional risk factors, provide additional information about the presence of coronary lesion^9^ and the risk of myocardial infarction, stroke, and CVD mortality.^10–13^ Glycosylation is the most abundant and diverse form of post-transcriptional modification, which participates in every physiological process.^14^ Protein glycosylation is driven by specific enzymes, and the complex carbohydrates (glycans) attached to, for example, immunoglobulins, have a specific regulatory role and result in differences in immune function.^15,16^ An altered protein glycosylation pattern has been described as a significant event that occurs during the transition from healthy to diseased tissue.^14,17^ This type of protein glycosylation is related to disease development in many syndromes, such as congenital disorders of glycosylation, cancer, inflammatory bowel diseases, renal disease, rheumatoid arthritis, chronic obstructive pulmonary disease, and AIDS.^18^ Some of the most important interactions between the immune system and pathogens are mediated by protein–glycan interactions, and it has been shown that alterations of the glycosylation of IgG, the most abundant immunoglobulin in circulation, have direct impact on its inflammatory properties.^16^ Different IgG glycosylation profiles may provide an at-risk phenotype to the developing of CVD because inflammation is known to play a crucial rule in CVD development.^19^ A study of 27 941 participants of the Women’s Health Study has previously shown that GlycA (glycoprotein acetylation), a biomarker of plasma protein glycan N–acetyl methyl groups (located on specific glycan branches of particular plasma proteins mainly α1 acid glycoprotein, haptoglobin, α1 antitrypsin, α1 antichymotrypsin, and transferrin), is related to incident CVD,^17^ which remained significant when adjusting for traditional risk factors and for C-reactive protein levels.^17^ GlycA, as a measure of protein glycosylation, has also been found to correlate with longitudinal risk of CVD and mortality in various cohort studies.^20^ However, besides GlycA, a large number of protein glycosylation traits can be measured.^21,22^ We hypothesized that these traits may reveal important information on the relationship between protein glycosylation, traditional risk factors, and subclinical atherosclerosis.

The aim of this study is to investigate the role of 76 IgG glycosylation traits in the risk of CVD measured with the 10-year ASCVD risk score^7^ by analyzing the IgG glycome composition in a large population-based female cohort from the United Kingdom (TwinsUK). We then replicated the significant results in an independent sample from the ORCADES cohort (Orkney Complex Disease Study). Finally, we investigate the association between the replicated glycan traits associated with CVD risk and presence of carotid and femoral atherosclerotic plaques in a subset of female individuals from the TwinsUK cohort.

## Methods

The TwinsUK data that support the findings of this study are publicly available on request on the department website (http://www.twinsuk.ac.uk/data-access/accessmanagement/). To access the ORCADES data, please email jim.wilson @ed.ac.uk.

### Discovery Cohort

Study subjects were individuals enrolled in the TwinsUK registry, a national register of adult twins.^23^ In this study, we analyzed data from 2970 females, 40 to 79 years old and without CVD. They had glycomics data available and the 10-year ASCVD risk score. The study was approved by St. Thomas’ Hospital Research Ethics Committee, and all twins provided informed written consent.

### Replication Cohort

The replication sample was drawn from the ORCADES. ORCADES is a family-based, cross-sectional study that seeks to identify genetic factors influencing cardiovascular and other disease risk in the isolated archipelago of the Orkney Isles in Northern Scotland.^24^ 2078 participants aged 16 to 100 years were recruited between 2005 and 2011, all of them having at least 2 Orcadian grandparents. Fasting blood samples were collected, and many health-related phenotypes and environmental exposures were measured in each individual. Here, we included 967 females with glycomics data available and the 10-year ASCVD risk score. All participants gave written informed consent, and the study was approved by Research Ethics Committees in Orkney and Aberdeen.

In addition to the replication performed in women, we further validated our results in 189 men from TwinsUK and 656 men from ORCADES.

### Phenotype Definitions

Data relevant to the present study include body mass index (body weight in kilograms divided by height in meter squared), T2D (defined as fasting glucose ≥7 mmol/L or physician’s letter confirming diagnosis), smoking (defined as current smoker and nonsmoker), treated and untreated systolic blood pressure, total and HDL cholesterol, and insulin. Fasting insulin levels were measured using the same methods as previously described.^25^ The homeostasis model assessment–estimated insulin resistance was calculated multiplying overnight fasting plasma insulin by overnight fasting plasma glucose, then dividing by the constant 22.5, ie, homeostasis model assessment–estimated insulin resistance=(fasting plasma insulin×fasting plasma glucose)/22.515. The ASCVD risk score is an algorithm used to estimate the 10-year cardiovascular risk of an individual using the individual’s sex, ethnicity, age, smoking status, cholesterol levels, blood pressure, and diabetes mellitus status.^7^ The individual risk of CVD was estimated using the 10-year ASCVD risk score.^7^

#### Femoral and Carotid Plaque

Left and right carotid and femoral arteries were visualized with B-mode ultrasound (Siemens CV70; Siemens, Erlangen, Germany, with 13-MHz vascular probe) as previously described.^26^ Briefly, arterial walls were examined for plaque in the common carotids, carotid bifurcations, origins of the internal and external carotid arteries, common femoral arteries, femoral bifurcations, and the origins of the superficial and deep femoral arteries. Plaque was defined in the longitudinal view as focal widening and protrusion into the lumen of ≥1.5-mm thickness relative to neighboring areas and confirmed in transverse view, and it was graded according to echogenicity.

### Analysis of IgG Glycans

IgG glycans were measured by Genos, Ltd, as previously described.^27,28^ Briefly, the IgG was isolated using protein G monolithic plates (BIA Separations, Ajdovščina, Slovenia). Dried IgG was denatured with 1.33% SDS (wt/vol), and N-glycans were released by digestion with PNGase F (ProZyme, Hayward, CA). After deglycosylation, N-glycans were labeled with 2-aminobenzamide fluorescent dye.

Free label and reducing agent were removed from the samples using hydrophilic interaction chromatography–solid-phase extraction.

Fluorescently labeled N-glycans were separated by hydrophilic interaction chromatography on a Waters Acquity UPLC instrument (Waters, Milford, MA). Data processing was performed using an automatic processing method with a traditional integration algorithm after which each chromatogram was manually corrected to maintain the same intervals of integration for all the samples. The chromatograms were all separated in the same manner into 24 peaks, and the amount of glycans in each peak was expressed as percentage of total integrated area. In addition to 24 directly measured glycan structures, 52 derived traits were calculated, which is a maximal number of traits we were able to calculate. These derived traits average particular glycosylation features (galactosylation, fucosylation, bisecting N-acetylglucosamine [GlcNAc], and sialylation; Online Table I). The derived glycan traits are calculated from directly measured glycans, and, therefore, their measurement error is smaller (at least the random error).^27^

### Lipoprotein Profiling and Glycoprotein by Nuclear Magnetic Resonance

Glycoprotein (GlycA), lipoproteins, and triglycerides were measured by Nightingale Health (previously known as Brainshake, Ltd, Finland; https://www.brainshake.fi/) from fasting serum samples using 500 MHz proton nuclear magnetic resonance spectroscopy as previously described.^29^

### Statistical Analysis

Statistical analysis was performed using Stata version 12 and R version 3.3.3.

Glycans were global normalized and log transformed because of right-skewness of their distributions. To remove experimental biases, all measurements were adjusted for batch and run-day effects using ComBat (R-package sva). Derived glycan traits were calculated using normalized and batch-corrected glycan measurements (exponential of batch-corrected measurements). All variables were centered and scaled to have SD 1. Outliers (>6 SD from the mean) were excluded from the analysis.

In the discovery cohort, association analyses between the 10-year ASCVD risk score and glycan traits were performed using linear mixed models adjusting for age, body mass index, and family relatedness as random effect. We used a conservative Bonferroni correction to account for multiple testing assuming 76 independent tests thus giving a significant threshold of (*P*<6.5×10^−^^4^=0.05/76). The Bonferroni significant 10-year ASCVD risk score glycan associations were replicated in 967 females from the ORCADES.

To adjust for kinship in the ORCADES cohort, the 10-year ASCVD risk score traits were set to their grammar+residuals in GenABEL using the genomic relationship matrix and no other covariates. These residuals are suitable for analysis as an unrelated population.^30^ These kinship-adjusted 10-year ASCVD risk score traits were then taken forward using the same (fixed only) effect model as TwinsUK. We then combined the results using inverse-variance fixed-effect meta-analysis.

Linear mixed model adjusting for covariates and family relatedness were then undertaken in the TwinsUK sample to determine the association between the identified glycan traits with the contributing factors of the 10-year ASCVD risk score (ie, T2D, smoking, total and HDL cholesterol, and systolic blood pressure) and with homeostasis model assessment.

We also looked at the association between the identified glycan traits with carotid and femoral plaque in a subset of 1382 female individuals from TwinsUK with plaque measured.

Finally, we created a glycan risk score in females from TwinsUK to assess the combined effects of all glycan traits identified. We fitted a logistic regression model for the significantly replicated glycans to a binary trait of high 10-year ASCVD risk score. For this, we selected the top quintile (corresponding to 10-year ASCVD risk score >5.2%) taking the *Z* scores of all the significant IgG glycans using both linear and quadratic terms and using a stepwise regression approach to account for the collinearities between glycan traits. The proportion of the variance in the 10-year ASCVD risk score was then assessed in women from TwinsUK and in men and women from ORCADES. The GlycA measure was added to the glycans from the score, and this IgG+GlycA was tested for association with carotid and femoral plaque adjusting for log 10-year ASCVD risk score.

## Results

**Figure 1.**
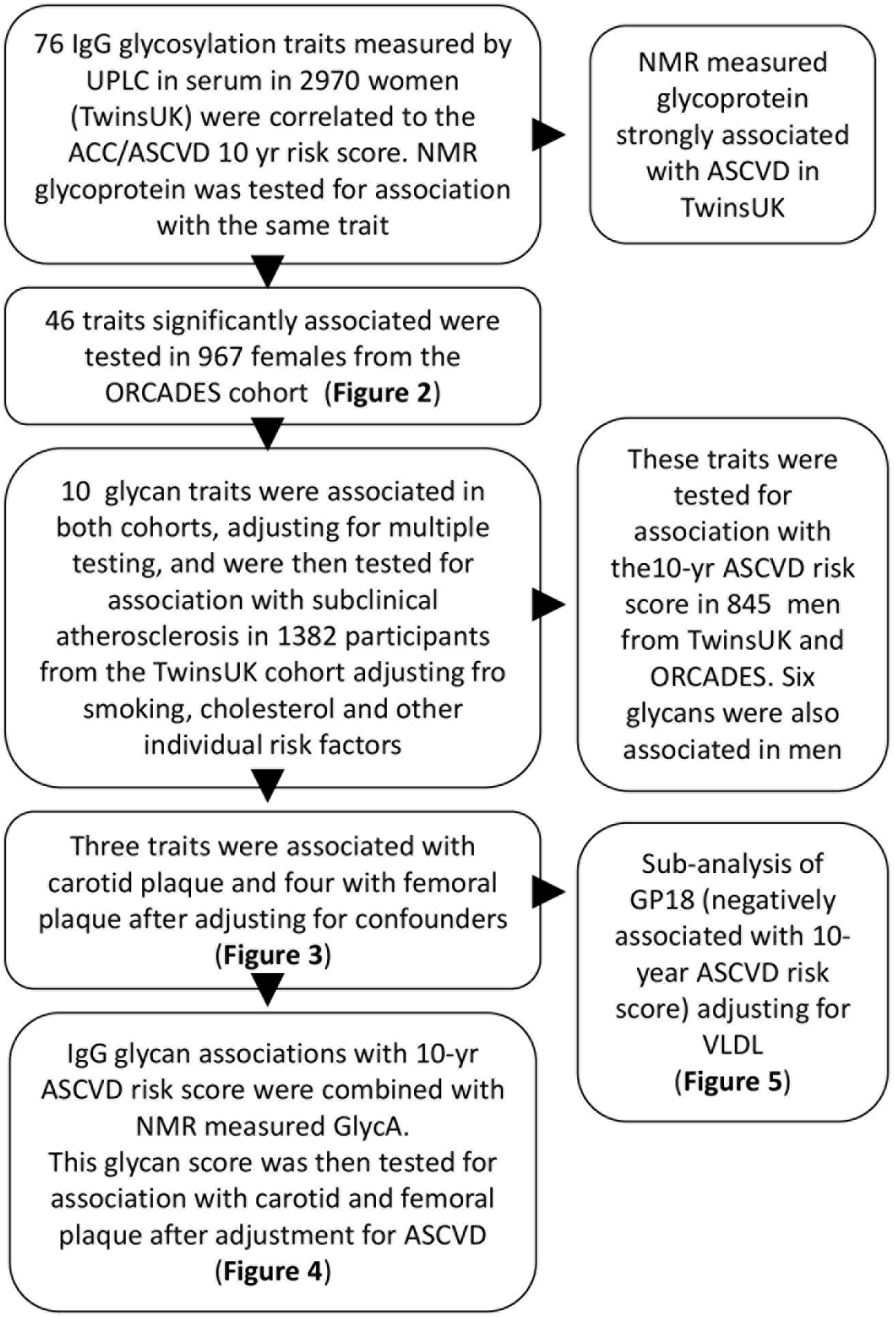
**Discovery: the role of glycan traits on cardiovascular risk estimates was tested on 3281 samples available.** Having identified traits significantly associated with cardiovascular disease risk, we replicated them first, in an independent cohort, validated them in men, and then investigated whether any of these associations could be exclusively explained by any of the individual factors that constitute the ACC/AHA 10-y atherosclerotic cardiovascular disease (ASCVD) risk estimate. The traits that remained associated were then tested for association with presence of subclinical atherosclerosis adjusting for the potential confounders. A subanalysis was performed for the IgG glycan GP18 (% of FA2G2S1 glycan among IgG where FA2G2S1 is the 2-AB mono-sialylated-, galactosylated biantennary N-glycan, core-substituted with fucose), which is strongly negatively correlated with very-low-density lipoprotein (VLDL). ACC indicates American College of Cardiology; AHA, American Heart Association; GP, glycan peak; GlycA, glycoprotein acetylation; ORCADES, Orkney Complex Disease Study; NMR, nuclear magnetic resonance; and UPLC, ultra performance liquid chromatography.

Levels of 76 IgG glycans (24 directly measured and 52 derived traits; Online Table I) were obtained in 2970 females from the TwinsUK sample and in 967 females from the ORCADES cohort with the American College of Cardiology/American Heart Association (ACC/AHA) ASCVD risk score available (age range, 40–79 years). The demographic characteristics of the study populations are presented in the Table. A flowchart of the study design is presented in Figure 1.

**Table.**
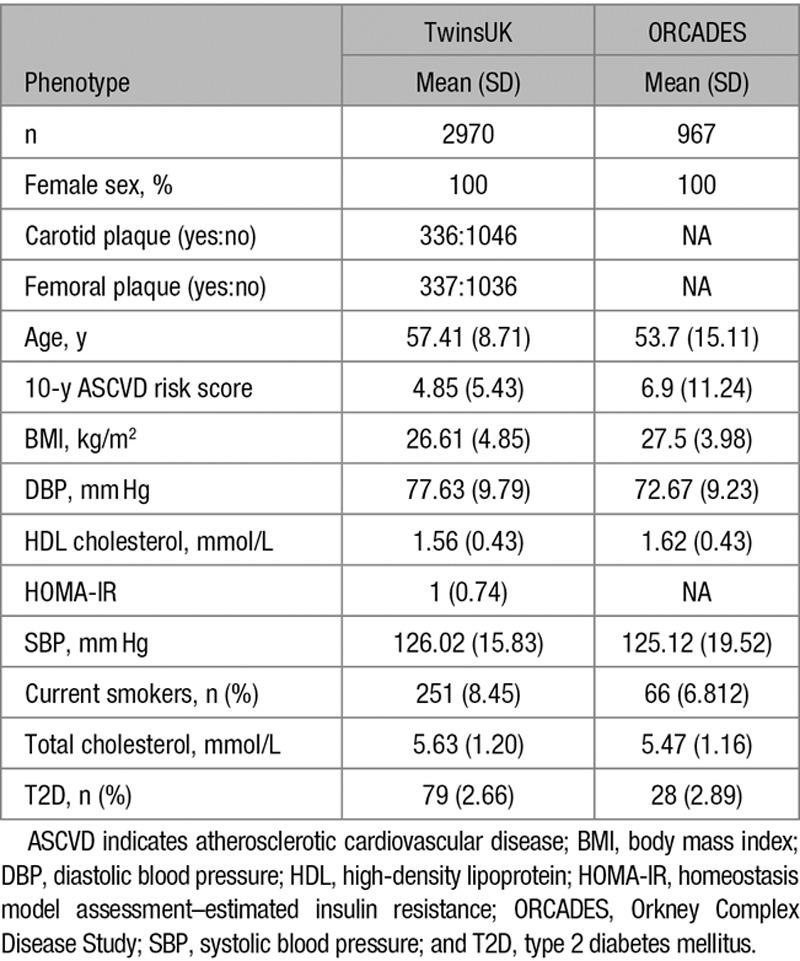
Demographic Characteristics of the Study Populations

### Discovery and Replication in Women

**Figure 2.**
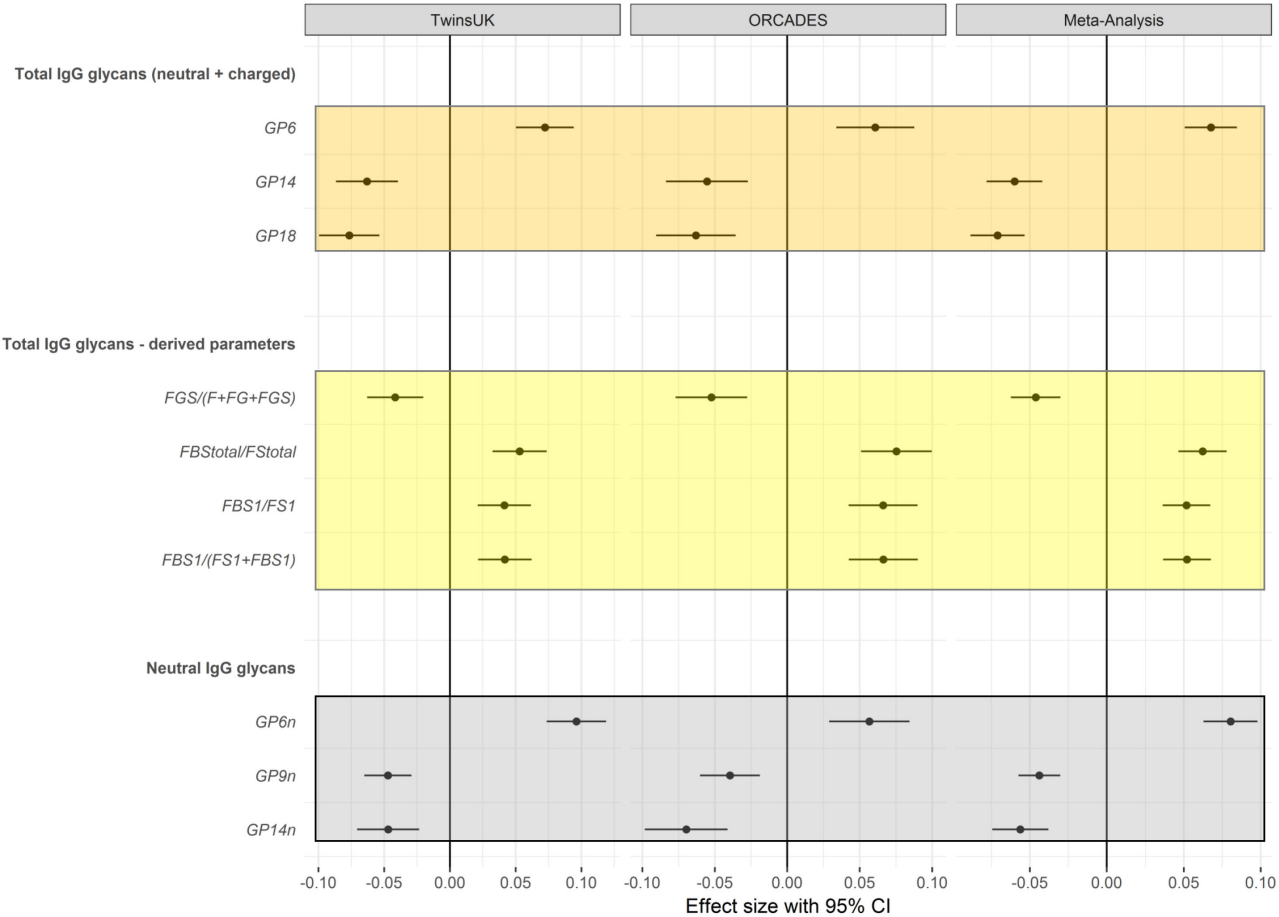
**Glycan traits significantly associated with ACC/AHA 10-y atherosclerotic cardiovascular disease risk score in the discovery, replication, and meta-analysis.** Analyses adjusted by age, sex, body mass index, family relatedness, and multiple testing. ACC indicates American College of Cardiology; AHA, American Heart Association; CI, confidence interval; FBS, sialylated fucosylated structures with bisecting GlcNAc; FG, fucosylated galactosylated structures without bisecting GlcNac; FGS, sialylated fucosylated galactosylated structures without bisecting GlcNAc; FS, sialylated fucosylated structures without bisecting GlcNAc; and GP, glycan peak.

We first ran linear mixed models in the discovery sample adjusting for age, body mass index, and family relatedness. We controlled for multiple testing using Bonferroni correction (*P*<6.58×10^−^^4^=0.05/76 glycan traits). This identified 46 glycan traits significantly associated with the 10-year ASCVD risk score; 25 glycan traits were positively associated with the 10-year ASCVD risk score, whereas 21 were negatively associated (Online Table I). We then assessed whether these associations with the 10-year ASCVD risk score were robust by testing for association these 46 glycans in 967 females from the ORCADES study. Out of those, 24 glycan traits were nominally associated with the 10-year ASCVD risk score (*P*<0.05) in the replication cohort, and 10 glycans were significantly associated with the 10-year ASCVD risk score after adjusting for covariates and multiple testing using Bonferroni correction (*P*<0.05/46). We then combined the results using inverse-variance fixed-effect meta-analysis (Figure 2).

### Validation in Men

We tested whether these results discovered in women and replicated in women were also associated in men. We find that 6 of the 10 glycans are also significantly associated in men when we meta-analyze IgG glycan data from ORCADES and TwinsUK (n=845; Online Table II and Online Figure I).

### Adjustment for Risk Factors in Women

**Figure 3.**
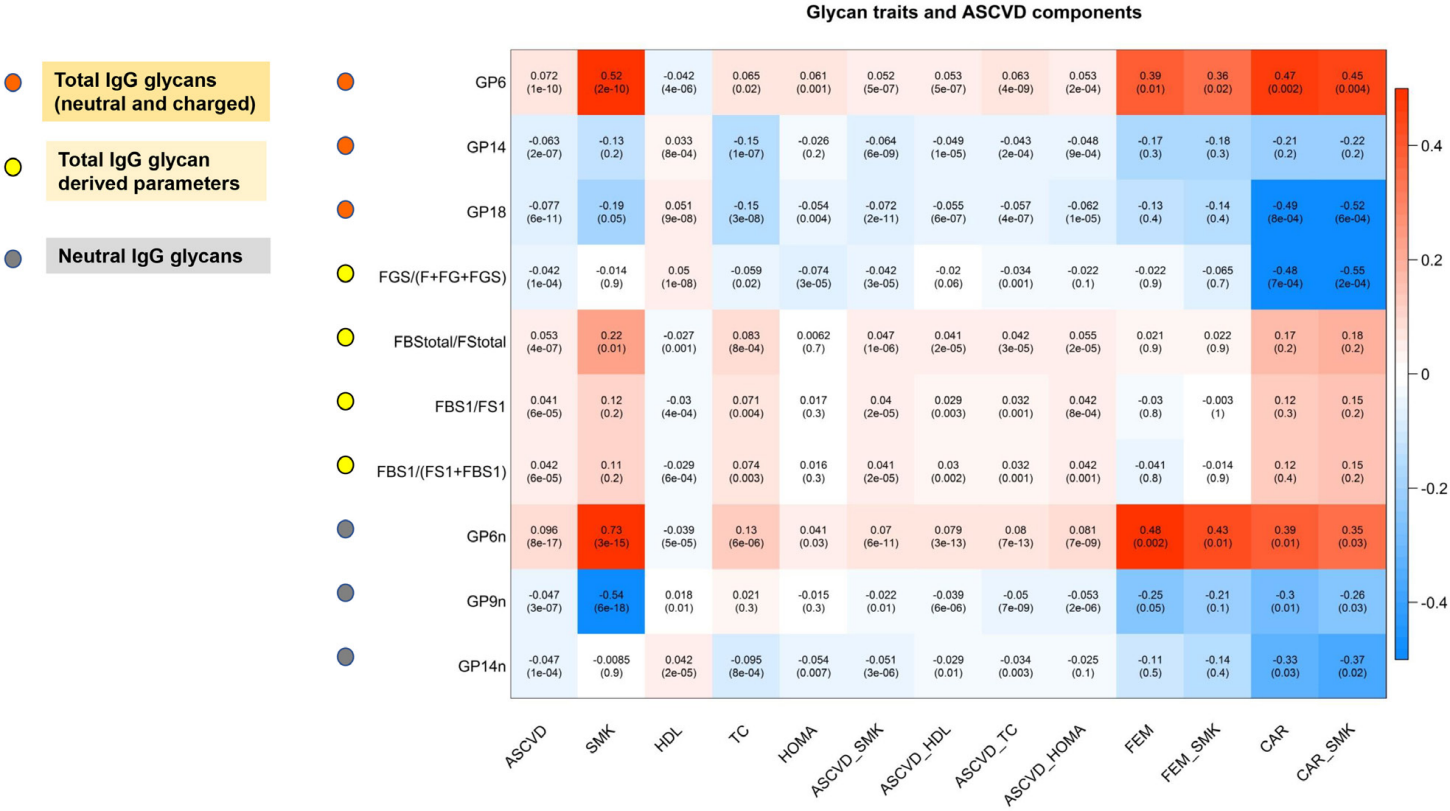
**Glycan traits and atherosclerotic cardiovascular disease (ASCVD) components**. Each cell of the matrix contains the regression coefficient between one glycan trait and a component of the 10-y ASCVD risk score and the corresponding *P* value. The table is color coded by correlation according to the table legend (red for positive and blue for negative correlations). ASCVD indicates 10-y ASCVD risk score; ASCVD_HDL, 10-y ASCVD risk score adjusted for covariates and HDL cholesterol; ASCVD_HOMA, 10-y ASCVD risk score adjusted for covariates and insulin resistance; ASCVD_SMK, 10-y ASCVD risk score adjusted for covariates and smoking; ASCVD_TC, 10-year ASCVD risk score adjusted for covariates and TC; CAR, carotid plaque; CAR_SMK, CAR adjusted for covariates and smoking; FEM, femoral plaque; FEM_SMK, FEM adjusted for covariates and smoking; HDL, high-density lipoprotein; HOMA, homeostasis model assessment insulin resistance; SMK, smoking; and TC, total cholesterol.

We investigated in TwinsUK women the association of the 10 replicated glycan traits with HDL and total cholesterol, smoking, systolic blood pressure, T2D, and insulin resistance. Although no associations were observed with T2D and systolic blood pressure (Online Table III), at *P*<0.05, all the glycan traits were associated with HDL cholesterol, 9 were associated with total cholesterol, 5 were associated with smoking, and 5 were associated with insulin resistance (Figure 3; Online Table III). After adjusting for the contributing risk factors (Figure 3; Online Table IV), we find that 8 of the associations always remain statistically significant.

### Association With Subclinical Atherosclerosis

We assessed in TwinsUK women the association between the glycan traits identified as associated with CVD risk after adjustment for individual risk factors and carotid and femoral plaque, which are well-known markers of subclinical coronary atherosclerosis.^9^ We find that 3 of these 8 glycan traits are associated with femoral plaque (*P*<0.05) and 4 of them are associated with carotid plaque (*P*<0.05), indicating that indeed these glycan traits are related to atherosclerosis. All but one of these associations remained significant (*P*<0.05) after adjusting for smoking (Figure 3; Online Table V).

### GlycA Nuclear Magnetic Resonance Association With the ACC/AHA 10-Year ASCVD Risk Score

Because many authors^31–33^ have shown the effect of nuclear magnetic resonance (NMR)-measured glycoprotein on cardiovascular mortality, we then investigated the association between this marker (GlycA) and ASCVD risk score. We find that indeed circulating levels of GlycA are positively and significantly correlated with the 10-year ASCVD risk score (0.14 [0.02]; *P*=8.49×10^−^^15^) in the TwinsUK cohort. Higher circulating levels of GlycA are also associated with a higher risk of developing both carotid (odds ratio [SE], 1.41 [0.21]; *P*=0.020 and femoral 1.57 [0.26]; *P*=0.005) plaques.

### Correlation Between GlycA and IgG Glycans

The NMR-measured GlycA shows a significant correlation with all the 8 glycan traits that are reproducibly associated with the 10-year ASCVD risk score (summarized in Online Table VI). However, the correlation is not large explaining 6% of variation in any of the IgG CVD-associated glycan trait. IgG glycan associations with the 10-year ASCVD risk score are consistent if we further adjust for GlycA.

#### Glycan Score

To assess the combined effects of all glycan traits, we fitted a logistic regression model of the 8 glycans (with and without GlycA) to the top quintile corresponding of the 10-year ASCVD risk score (>5.2%) to compute a linear glycan score in females from the TwinsUK cohort (in which women have both UPLC and NMR measures). After stepwise regression, the model fitted on standardized (mean zero, variance 1, ie, *Z* scores) of the IgG glycan measures was IgG score:


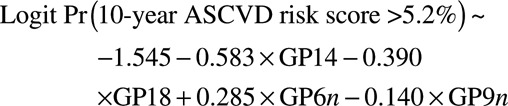
(1)

This linear combination was associated with log (10-year ASCVD risk score) in a linear regression with β (SE)=0.477 (0.0211), *P*=2.3×10^−^^96^ explaining 26.9% of the variance in log(10-year risk ASCVD score) in our data. This score was then tested for association with log(10-year ASCVD risk score) in ORCADES where it explained 54.6% of the variance in log(10-year ASCVD risk score; β [SE]=0.412 [0.012]; *P*=5.1×10^−^^168^) in women and 39.5% of the variance in log (10-year risk ASCVD score; β [SE]=0.443 [0.021]; *P*=1.7×10^−^^73^) in men.

We then adjusted for GlycA levels in TwinsUK (where the NMR measure was available) the role of this glycan score. Adjusting for GlycA resulted in an association for the IgG score with β (SE)=0.441(0.0211) *P*=4.4×10^−^^84^ and of β (SE)=0.193 (0.023) *P*=5.5×10^−16^ for GlycA, indicating a significant contribution for both the combined IgG glycans and NMR measure.

We, therefore, computed a glycan score based on both IgG glycans plus GlycA. The model identified was IgG+GlycA score:


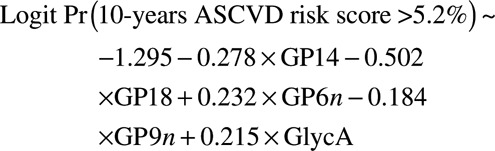
(2)

This measure is strongly associated with log (10-year ASCVD risk score; β [SE]=0.571 [0.023]; *P*=2.1×10^−^^110^) and explains 30.1% of the variation in log (10-year ASCVD risk score) in the TwinsUK data.

**Figure 4.**
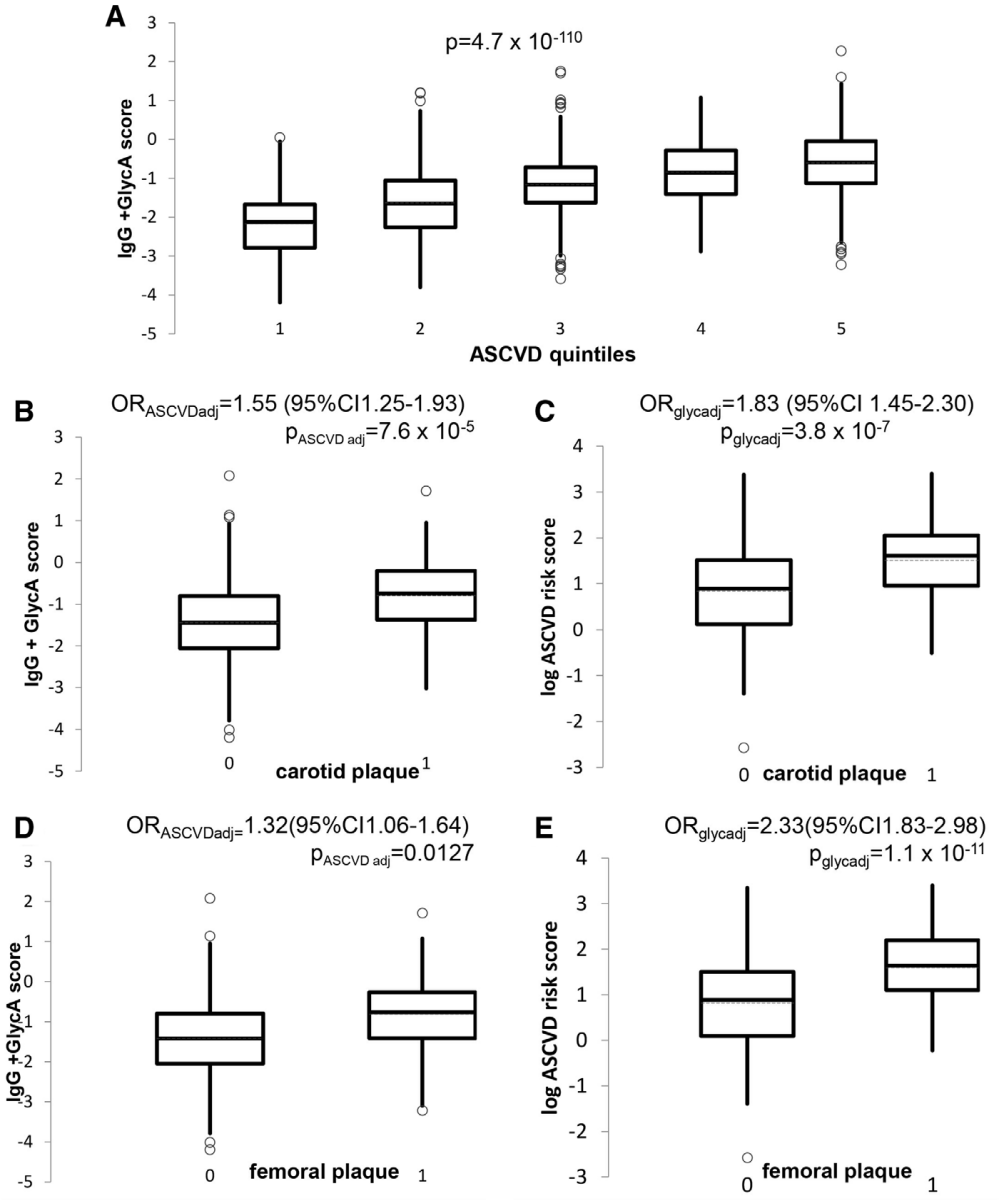
**Combined glycan score vs cardiovascular disease risk and measures of subclinical atherosclerosis. A**, Box plot showing the distribution of the glycan score in quintiles of the 10-y atherosclerotic cardiovascular disease (ASCVD) risk score. **B**, Box plot showing the distribution of the glycan (IgG+GlycA) score in individuals with and without carotid plaque. *P* values and odds ratios (OR) from logistic regression adjusted for log (10-y ASCVD risk score). **C**, Box plot showing the distribution of the log (10-y ASCVD risk score) in individuals with and without carotid plaque, OR and *P* value adjusted for glycan score. **D**, Box plot showing the distribution of the glycan score in individuals with and without femoral plaque. *P* value and OR adjusted for log (10-y ASCVD risk score). **E**, Box plot showing the distribution of the log (10-y ASCVD risk score) in individuals with and without femoral plaque, OR and *P* value adjusted for glycan score. CI indicates confidence interval.

The distribution of this IgG+GlycA score for each of the 5 quintiles of the 10-year ASCVD risk score distribution is shown as box plots in Figure 4A. We proceeded to compare the association between the glycan score and 10-year ASCVD risk score on subclinical atherosclerosis. The associations of the glycan score in individuals with carotid and femoral plaque are presented in Figure 4B and 4D, whereas the distribution of log (10-year ASCVD risk score) in the same individuals is depicted in Figure 4C and 4E.

In quantitative terms, the association between the glycan score and carotid plaque—adjusting for the 10-year ASCVD risk score—is OR=1.55; 95% confidence interval, 1.25–1.93; *P*=7.5×10^−^^5^, whereas the 10-year ASCVD risk score (adjusted for the glycan score) is associated with OR=1.83; 95% confidence interval, 1.45–2.30; *P*=3.8×10^−^^7^. For femoral plaque, the association of the glycan score (adjusted for the 10-year ASCVD risk score) was OR=1.32; 95% confidence interval, 1.06–1.64; *P*=0.01 and that of the 10-year ASCVD risk score (adjusted for glycan score) was OR=2.33; 95% confidence interval, 1.83–2.98; *P*=1.1×10^−^^11^. Thus, the glycan score contributes significantly to both measures of subclinical atherosclerosis in addition to the known CVD risk factors.

### GP18 and Very-Low-Density Lipoprotein

**Figure 5.**
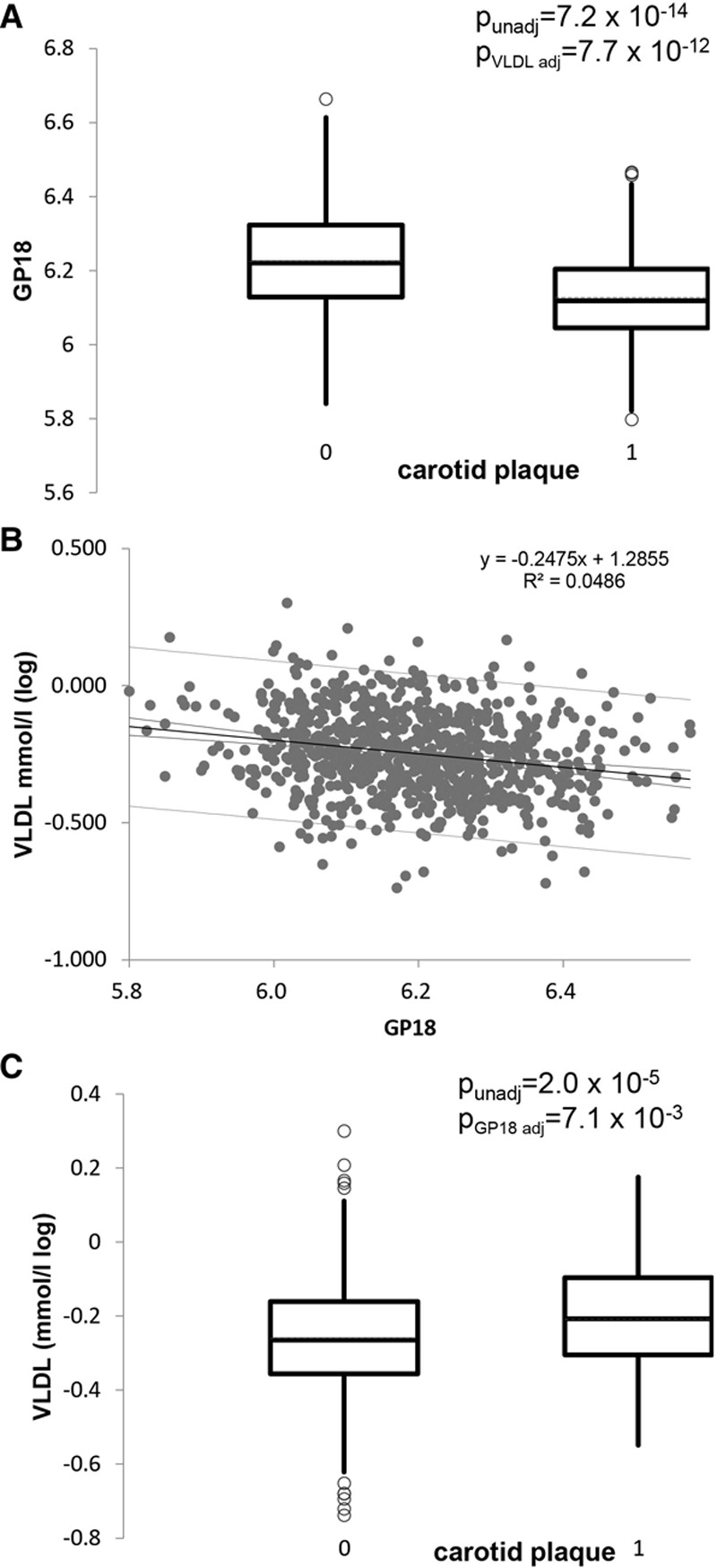
**Association between GP18 (% of FA2G2S1 glycan among IgG where FA2G2S1 is the 2-AB mono-sialylated-, galactosylated biantennary N-glycan, core-substituted with fucose), carotid plaque and circulating levels of VLDL. A**, Box plot showing the distribution of the IgG glycan trait GP18 in individuals with and without carotid plaque. The *P* values shown are unadjusted and adjusted for circulating levels of very-low-density lipoprotein (VLDL). **B**, Correlation between circulating VLDL and GP18. **C**, Distribution of VLDL in individuals with and without carotid plaque. The *P* values shown are unadjusted and adjusted for levels of GP18.

One of the glycan traits, GP18 (FA2G2S1), is negatively associated with ASCVD risk score, total cholesterol, and carotid plaque; however, the association remains significant after adjusting for total cholesterol. We, therefore, investigated its relationship to other measures of lipoproteins and triglycerides using the Nightingale platform. This monosialylated glycan with core fucose is strongly negatively correlated with various measures of lipids and triglycerides, in particular with the concentration of very-low-density lipoprotein (VLDL) and triglycerides in VLDL (Online Table VII). To illustrate the magnitude of the associations between carotid plaque and glycosylation traits, the distribution of GP18 in individuals with and without carotid plaque is shown in Figure 5 side by side to the distribution of 10-year ASCVD risk score.

## Discussion

In this study, we report that there are significant and reproducible IgG glycan traits associated with cardiovascular risk in addition to the previously reported ones with one single measure of protein glycosylation (GlycA). After adjustment for individual risk factors, we identify 8 quantitative IgG glycan traits associated with the 10-year ASCVD risk score in women from 2 independent cohorts, 6 of which are also associated in men. Four of the glycan traits identified are also associated with presence of subclinical atherosclerosis after adjusting for all traditional risk factors (3 with both femoral and carotid plaques and 1 with carotid plaque only), indicating that indeed these glycan traits are related to atherosclerosis.

Several recent studies have used targeted metabolomics platforms to examine a glycan signal (referred to as GlycA) thought to identify the concentration of circulating protein-bound N-acetyl methyl groups of GlcNAc and N-acetylgalactosamine glycan moieties based on NMR measures.^33^ One such study^33^ demonstrated that this signal was associated with longitudinal risk of mortality related to both ASCVD and cancer.^34^

In this study, we have used the NMR GlycA measure as a positive control and find that this measure of protein glycosylation previously reported to be associated with CVD mortality by Lawler et al^33^ is also strongly associated with our data. In addition, 8 of the glycan associations we report to be associated with 10-year ASCVD risk score remain significant after adjusting for individual risk factors, indicating that they are independently contributing to CVD risk. This is line with literature data indicating that IgG glycans are only weakly related to the GlycA NMR signal.^35^ Importantly, we find that a linear combination of IgG glycans predicts a large proportion of the variance in 10-year ASCVD risk score both in men (39%) and in women (26% to 54%) and that this is reproducible.

When we combined the GlycA NMR measure into an IgG+GlycA score, we found that it was strongly associated with both femoral and carotid plaques in the TwinsUK cohort. The association with subclinical atherosclerosis of this measure remained statistically significant after adjustment for 10-year ASCVD risk score, in line with previous reports^20^ that measures of protein glycosylation contribute to cardiovascular risk in addition to traditional risk factors. It is of interest that the association between the IgG glycan traits and CVD-related study end points remain significant after adjusting for GlycA, suggesting that incremental information is gained with the different measures of glycans.

It has been hypothesized^20^ that protein glycosylation may be capturing a combined measure of upstream inflammation related to risk of ASCVD. Recently, there has been an emerging focus on reclassification of diseases based on common mechanisms of pathophysiology and away from traditional clinical manifestation–defined approaches.^36^ Our data, with more comprehensive measures of protein glycosylation, highlight the potential value of glycomics in identifying such pathways of disease, the reproducibility of results across different cohorts, and the extent to which CVD risk can be captured by these measures.

We also report that some IgG glycans are associated with higher CVD risk and others are associated with lower CVD risk. More precisely, glycans that contain exposed 3 GlcNAcs (GP6) or glycans that contain both bisecting GlcNAc and 1 sialic acid are positively associated with CVD risk (consistent with the previous GlycA reports), whereas sialylated glycans without a bisecting GlcNAc are negatively associated. Increased levels of glycans with a bisecting GlcNAc are reported to associate with higher age, whereas decreased levels were associated with longevity.^37^ Even though only nongalactosylated glycoforms with a bisecting GlcNAc were associated with familial longevity, our results show that association of bisecting GlcNAc and CVD risk is not dependent on the presence of other sugar residues^27,37^; we found agalactosylated, monogalactosylated, and sialylated N-glycans with a bisecting GlcNAc positively associated with CVD risk. Besides aging, increased levels of glycans with a core fucose and bisecting GlcNAc are known to be present in serum of patients with T2D,^38^ and most of these glycans are coming from IgG,^39^ thus reflecting the same traits connected with CVD risk in this study.

Age, T2D, and smoking are all factors included in the 10-year ASCVD cardiovascular risk score assessment and have a positive association with a bisecting GlcNAc. IgG glycosylation is able to modulate Fc receptor binding, and bisecting GlcNAc was shown to increase antibody-dependent cellular cytotoxicity mediated by binding of the antibody to the Fcγ-receptor.^40^ Glycan traits known to increase antibody-dependent cellular cytotoxicity are involved in proinflammatory pathways,^41^ and inflammation is known to be underlying mechanism of CVD’s development.^19^

Although bisecting GlcNAc is related with proinflammatory activity of IgG and the aforementioned conditions, sialylation and core fucosylation are consistently associated with anti-inflammatory activity.^16,42^ Indeed, a core fucosylated digalactosylated monosialylated glycan, GP18 (also called FA2G2S1), over all glycan traits remains strongly associated with the 10-year ASCVD risk score after adjustment for the individual risk factors that constitute the ASCVD risk score. After further investigation, we found that this glycan structure is strongly negatively correlated with VLDL levels. VLDL itself is a risk factor for CVD being associated with hypertriglyceridemia and dyslipidemia in general.^43^ Importantly, defects in the cholesterol metabolism pathway (particularly in the generation of nonsterol isoprene compounds) lead to disturbances in the glycosylation of proteins. This suggests a functional link between cholesterol metabolism and protein glycosylation.^44^ Moreover, in rabbits, IgG and VLDL were shown to contribute to arterial lesions and that sialic acid plays a crucial role in the prevention of an arterial lesion formation^45^; even though our work supports that connection, the complete picture is still missing. Therefore, further studies should be performed focusing on the role of these glycosylated structures in predicting cardiovascular events and, in particular, their interaction with VLDL.

We note some study limitations. First, the results were discovered and replicated primarily in women, even though most of the results are also replicated in men. Second, the cross-sectional nature of our data does not allow us to draw conclusions as to whether the identified glycan traits are causative of CVD decline or merely correlated with it, although the results from these hypothesis-generating findings are consistent with other less comprehensive measures of glycosylations where causative links between glycosylation and CVD outcomes have been shown.^33,34^ Third, the associations were discovered with the 10-year ASCVD risk score and not with actual CVD events. We report that these associations were also validated with measures of subclinical atherosclerosis after adjusting for all the risk factors in the 10-year ACC/AHA risk score, aiming to show that glycan traits provide molecular information that was not present in the pooled risk equation and thus suggesting an important biological role for these post-translational IgG modifications.

In conclusion, our data point to separate pathways whereby immunoglobulin glycosylation may be related to cardiovascular risk; however, a large number of N-glycan traits related to core fucose and bisecting GlcNAc are strongly associated with atherosclerotic plaque.

However, one specific trait related to the sialylated N-glycan seems to be strongly negatively related to circulating VLDL and is supportive of a role of IgG glycosylation in VLDL metabolism and arterial lesion formation also in humans.

## Acknowledgments

We wish to express our appreciation to all study participants of the TwinsUK cohort. We want to acknowledge Dr Benyu Jiang for performing the majority of the carotid measurements in TwinsUK. Toma Keser, Jerko Štambuk, Mirna Šimurina, Tamara Pavić, and Jasminka Krištić are acknowledged for their help during the laboratory work on glycan analysis. We would like to acknowledge the invaluable contributions of the research nurses in Orkney, the administrative team in Edinburgh, and the people of Orkney.

## Sources of Funding

This work was funded by the British Heart Foundation (BHF) Special Project grant SP/12/4/29573 and Medical Research Council (MRC)/British Heart Foundation Ancestry and Biological Informative Markers for Stratification of Hypertension (AIMHY; MR/M016560/1) grant. Twins UK receives funding from the Wellcome Trust European Community’s Seventh Framework Programme (FP7/2007–2013 to TwinsUK); the National Institute for Health Research (NIHR) Clinical Research Facility at Guy’s & St Thomas’ National Health Service (NHS) Foundation Trust and NIHR Biomedical Research Centre based at Guy’s and St Thomas’ NHS Foundation Trust and King’s College London. This work also receives funding from the European Union’s Seventh Programme for research, technological development and demonstration under grant agreement No 603946 (Health and Environment-wide Associations Based on Large population Surveys (HEALS). Glycan analysis was supported by European Commission Framework programme 7 grants Methods for Integrated analysis of Multiple Omics datasets (MIMOmics; contract number 305280), H2020 grants GlySign (contract number 722095), and IMforFuture (contract number 721815), as well as by the European Structural and Investment Funds IRI (grant number KK.01.2.1.01.0003) and Croatian National Centre of Research Excellence in Personalized Healthcare (grant number KK.01.1.1.01.0010). ORCADES (Orkney Complex Disease Study) was supported by the Chief Scientist Office of the Scottish Government (CZB/4/276, CZB/4/710), the Royal Society, the MRC Human Genetics Unit quinquennial programme QTL in Health and Disease, Arthritis Research UK, and the European Union framework program 6 EUROSPAN project (contract number LSHG-CT-2006–018947). T.D. Spector is a National Institute for Health Research Senior Investigator. A.M. Valdes is supported by the NIHR Nottingham British Research Council.

## Disclosures

G. Lauc is a founder and owner, I. Gudelj and I. Trbojević-Akmačić are employees of Genos, Ltd, which offers commercial service of glycomic analysis and has 2 patents in this field (WO/2014/203010 and WO/2017/215973). The other authors report no conflicts.

## Supplementary Material

## Novelty and Significance

### What Is Known?

- Sugar molecules can attach to proteins in a process called glycosylation, which plays an important role in regulating inflammation.
- One of these proteins, GlycA, is involved with an increased risk of heart disease.
- There are many different types of glycosylated proteins, and overall, they constitute the glycome.
