# Supplementary material for: Glycosylation Profile of Immunoglobulin G Is Cross-Sectionally Associated With Cardiovascular Disease Risk Score and Subclinical Atherosclerosis in Two Independent Cohorts
Source: Circ Res. 2018 Mar 13;122(11):1555–64. doi: 10.1161/CIRCRESAHA.117.312174 (PMC5970566; doi:10.1161/CIRCRESAHA.117.312174)

**Glycosylation profile of Immunoglobulin G is cross-sectionally associated with cardiovascular disease risk score and subclinical atherosclerosis in two independent cohorts**

Cristina Menni, PhD [a*], Ivan Gudelj, PhD[b*] , Erin MacDonald-Dunlop, MSc[c], Massimo Mangino, PhD [a], Jonas Zierer, PhD [a], Erim Besić, PhD[d], Peter K Joshi, PhD[c], Irena Trbojević-Akmačić, PhD[b] Phil Chowienczyk, MD[d] , Tim D Spector, MD [a], James F Wilson, PhD[c,e], Gordan Lauc, PhD [b,f**]Ana M Valdes, PhD [a, g,h**]

[a] Department of Twin Research and Genetic Epidemiology, King's College London, London, UK.

[b] Genos Glycoscience Research Laboratory, Zagreb, Croatia

[c] Centre for Global Health Research, Usher Institute of Population Health Sciences and Informatics, University of Edinburgh, Teviot Place, Edinburgh, EH8 9AG, Scotland

[d]Department of Clinical Pharmacology, King's College London, London, UK.

[e]MRC Human Genetics Unit, Institute of Genetics and Molecular Medicine, University of Edinburgh, Western General Hospital, Edinburgh, EH4 2XU, Scotland

[f] University of Zagreb, Faculty of Pharmacy and Biochemistry, Zagreb, Croatia

[g]School of Medicine, Nottingham City Hospital, Hucknall Road, Nottingham, UK.

[h] NIHR Nottingham Biomedical Research Centre, Nottingham, UK.

***Supplementary Material***

**OnlineTable I. Description of 24 quantitative IgG glycosylation traits and 52 derived trait and association between all tested glycans and derived traits with the 10-year ASCVD risk score in the discovery cohort. Analyses adjusted by age, BMI and family relatedness. Significance cut-off: P< $6.58x10^{-4}$**

| | | | | **10-year ASCVD risk score** | |
|---|---|---|---|---|---|
| **GROUP** | **Glycan** | **DESCRIPTION*** | **FORMULA*** | **Beta(SE)** | **P** |
| *Total IgG glycans (neutral + charged)* | GP1 | *The percentage of FA1 glycan in total IgG glycans* | *GP1 / GP* 100* | 0.002(0.01) | 8.46E-01 |
| | GP2 | *The percentage of A2 glycan in total IgG glycans* | *GP2 / GP* 100* | 0.051(0.01) | 3.45E-07 |
| | GP4 | *The percentage of FA2 glycan in total IgG glycans* | *GP4 / GP* 100* | -0.005(0.011) | 6.36E-01 |
| | GP5 | *The percentage of M5 glycan in total IgG glycans* | *GP5 / GP* 100* | 0.037(0.01) | 1.53E-04 |
| | GP6 | *The percentage of FA2B glycan in total IgG glycans* | *GP6 / GP* 100* | 0.072(0.011) | 1.30E-10 |
| | GP7 | *The percentage of A2G1 glycan in total IgG glycans* | *GP7 / GP* 100* | 0.028(0.009) | 2.52E-03 |
| | GP8 | *The percentage of FA2[6]G1 glycan in total IgG glycans* | *GP8 / GP* 100* | -0.046(0.009) | 8.06E-07 |
| | GP9 | *The percentage of FA2[3]G1 glycan in total IgG glycans* | *GP9 / GP* 100* | -0.052(0.009) | 1.76E-08 |
| | GP10 | *The percentage of FA2[6]BG1 glycan in total IgG glycans* | *GP10 / GP* 100* | 0.048(0.009) | 1.93E-07 |
| | GP11 | *The percentage of FA2[3]BG1 glycan in total IgG glycans* | *GP11 / GP* 100* | 0.054(0.01) | 3.42E-08 |
| | GP12 | *The percentage of A2G2 glycan in total IgG glycans* | *GP12 / GP* 100* | 0(0.01) | 9.66E-01 |
| | GP13 | *The percentage of A2BG2 glycan in total IgG glycans* | *GP13 / GP* 100* | -0.01(0.009) | 2.80E-01 |
| | GP14 | *The percentage of FA2G2 glycan in total IgG glycans* | *GP14 / GP* 100* | -0.063(0.012) | 1.49E-07 |

| | | | | | |
|---|---|---|---|---|---|
| | GP15 | *The percentage of FA2BG2 glycan in total IgG glycans* | *GP15 / GP* 100* | 0.005(0.011) | 6.35E-01 |
| | GP16 | *The percentage of FA2G1S1 glycan in total IgG glycans* | *GP16 / GP * 100* | -0.049(0.009) | 1.32E-07 |
| | GP17 | *The percentage of A2G2S1 glycan in total IgG glycans* | *GP17/ GP * 100* | 0.012(0.009) | 1.84E-01 |
| | GP18 | *The percentage of FA2G2S1 glycan in total IgG glycans* | *GP18 / GP * 100* | -0.077(0.012) | 5.59E-11 |
| | GP19 | *The percentage of FA2BG2S1 glycan in total IgG glycans* | *GP19 / GP * 100* | -0.013(0.009) | 1.55E-01 |
| | GP20+GP21 | | *(GP20+GP21)/GP * 100* | 0.017(0.009) | 6.23E-02 |
| | GP22 | *The percentage of A2BG2S2 glycan in total IgG glycans* | *GP22 / GP * 100* | 0.022(0.009) | 8.87E-03 |
| | GP23 | *The percentage of FA2G2S2 glycan in total IgG glycans* | *GP23 / GP * 100* | -0.071(0.01) | 1.43E-13 |
| | GP24 | *The percentage of FA2BG2S2 glycan in total IgG glycans* | *GP24 / GP * 100* | -0.003(0.009) | 7.04E-01 |
| | FGS/(FG+FGS) | *The percentage of sialylation of fucosylated galactosylated structures without bisecting GlcNAc in total IgG glycans* | *SUM(GP16 + GP18 + GP23) / SUM(GP16 + GP18 + GP23 + GP8 + GP9 + GP14)* 100* | -0.034(0.01) | 5.36E-04 |
| | FBGS/(FBG+FBGS) | *The percentage of sialylation of fucosylated galactosylated structures with bisecting GlcNAc in total IgG glycans* | *SUM(GP19 + GP24) / SUM(GP19 + GP24 + GP10 + GP11 + GP15)* 100* | -0.031(0.009) | 4.26E-04 |
| | FGS/(F+FG+FGS) | *The percentage of sialylation of all fucosylated structures without bisecting GlcNAc in total IgG glycans* | *SUM(GP16 + GP18 + GP23) / SUM(GP16 + GP18 + GP23 + GP4 + GP8 + GP9 + GP14)* 100* | -0.042(0.011) | 1.31E-04 |
| *Total IgG glycans - derived parameters* | FBGS/(FB+FBG+FBGS) | *The percentage of sialylation of all fucosylated structures with bisecting GlcNAc in total* | *SUM(GP19 + GP24) / SUM(GP19 + GP24 + GP6 + GP10 + GP11* | -0.042(0.009) | 4.47E-06 |

| | | | | | |
|---|---|---|---|---|---|
| | | *IgG glycans* | *+ GP15)* 100* | | |
| | FG1S1/(FG1+FG1S1) | *The percentage of monosialylation of fucosylated monogalactosylated structures in total IgG glycans* | *GP16 / SUM(GP16 + GP8 + GP9)* 100* | -0.005(0.009) | 5.89E-01 |
| | FG2S1/(FG2+FG2S1+FG2S2) | *The percentage of monosialylation of fucosylated digalactosylated structures in total IgG glycans* | *GP18 / SUM(GP18 + GP14 + GP23)* 100* | -0.021(0.009) | 1.93E-02 |
| | FG2S2/(FG2+FG2S1+FG2S2) | *The percentage of disialylation of fucosylated digalactosylated structures in total IgG glycans* | *GP23 / SUM(GP23 + GP14 + GP18)* 100* | -0.027(0.009) | 2.99E-03 |
| | FBG2S1/(FBG2+FBG2S1+FBG2S2) | *The percentage of monosialylation of fucosylated digalactosylated structures with bisecting GlcNAc in total IgG glycans* | *GP19 / SUM(GP19 + GP15 + GP24)* 100* | -0.018(0.009) | 5.64E-02 |
| | FBG2S2/(FBG2+FBG2S1+FBG2S2) | *The percentage of disialylation of fucosylated digalactosylated structures with bisecting GlcNAc in total IgG glycans* | *GP24 / SUM(GP24 + GP15 + GP19)* 100* | -0.001(0.009) | 9.00E-01 |
| | $F^{total}S1/F^{total}S2$ | *Ratio of all fucosylated (+/- bisecting GlyNAc) monosialylated and disialylated structures in total IgG glycans* | *SUM(GP16 + GP18 + GP19) / SUM(GP23 + GP24)* | -0.009(0.009) | 3.29E-01 |
| | FS1/FS2 | *Ratio of fucosylated (without bisecting GlcNAc) monosialylated and disialylated structures in total IgG glycans* | *SUM(GP16 + GP18) / GP23* | 0.028(0.009) | 1.50E-03 |

| | | | | | |
|---|---|---|---|---|---|
| | FBS1/FBS2 | *Ratio of fucosylated (with bisecting GlcNAc) monosialylated and disialylated structures in total IgG glycans* | *GP19 / GP24* | -0.01(0.009) | 2.65E-01 |
| | $FBS^{total}/FS^{total}$ | *Ratio of all fucosylated sialylated structures with and without bisecting GlcNAc* | *SUM(GP19 + GP24) / SUM(GP16 + GP18 + GP23)* | 0.053(0.01) | 4.18E-07 |
| | FBS1/FS1 | *Ratio of fucosylated monosialylated structures with and without bisecting GlcNAc* | *GP19 / SUM(GP16 + GP18)* | 0.041(0.01) | 6.08E-05 |
| | FBS1/(FS1+FBS1) | *The incidence of bisecting GlcNAc in all fucosylated monosialylated structures in total IgG glycans* | *GP19 / SUM(GP16 + GP18 + GP19)* | 0.042(0.01) | 5.97E-05 |
| | FBS2/FS2 | *Ratio of fucosylated disialylated structures with and without bisecting GlcNAc* | *GP24 / GP23* | 0.101(0.01) | 9.48E-23 |
| | FBS2/(FS2+FBS2) | *The incidence of bisecting GlcNAc in all fucosylated disialylated structures in total IgG glycans* | *GP24 / SUM(GP23 + GP24)* | 0.101(0.011) | 2.23E-21 |
| | $GP1^n$ | *The percentage of FA1 glycan in total neutral IgG glycans ($GP^n$)* | *GP1 / $GP^n$ * 100* | 0.009(0.01) | 3.48E-01 |
| | $GP2^n$ | *The percentage of A2 glycan in total neutral IgG glycans ($GP^n$)* | *GP2 / $GP^n$ * 100* | 0.055(0.01) | 3.34E-08 |
| | $GP4^n$ | *The percentage of FA2 glycan in total neutral IgG glycans ($GP^n$)* | *GP4 / $GP^n$ * 100* | 0.007(0.012) | 5.32E-01 |
| *Neutral IgG glycans* | $GP5^n$ | *The percentage of M5 glycan in total neutral IgG glycans ($GP^n$)* | *GP5 / $GP^n$ * 100* | 0.046(0.01) | 2.01E-06 |

| | | | | | |
|---|---|---|---|---|---|
| | $GP6^n$ | *The percentage of FA2B glycan in total neutral IgG glycans ($GP^n$)* | *$GP6 / GP^n * 100$* | 0.096(0.012) | 7.93E-17 |
| | $GP7^n$ | *The percentage of A2G1 glycan in total Ineutral IgG glycans ($GP^n$)* | *$GP7 / GP^n * 100$* | 0.032(0.009) | 5.28E-04 |
| | $GP8^n$ | *The percentage of FA2[6]G1 glycan in total neutral IgG glycans ($GP^n$)* | *$GP8 / GP^n * 100$* | -0.044(0.01) | 4.31E-06 |
| | $GP9^n$ | *The percentage of FA2[3]G1 glycan in total neutral IgG glycans ($GP^n$)* | *$GP9 / GP^n * 100$* | -0.047(0.009) | 2.58E-07 |
| | $GP10^n$ | *The percentage of FA2[6]BG1 glycan in total neutral IgG glycans ($GP^n$)* | *$GP10 / GP^n * 100$* | 0.062(0.009) | 1.99E-11 |
| | $GP11^n$ | *The percentage of FA2[3]BG1 glycan in total neutral IgG glycans ($GP^n$)* | *$GP11 / GP^n * 100$* | 0.067(0.01) | 4.19E-12 |
| | $GP12^n$ | *The percentage of A2G2 glycan in total neutral IgG glycans ($GP^n$)* | *$GP12 / GP^n * 100$* | 0.006(0.01) | 5.30E-01 |
| | $GP13^n$ | *The percentage of A2BG2 glycan in total neutral IgG glycans ($GP^n$)* | *$GP13 / GP^n * 100$* | 0.003(0.01) | 7.79E-01 |
| | $GP14^n$ | *The percentage of FA2G2 glycan in total neutral IgG glycans ($GP^n$)* | *$GP14 / GP^n * 100$* | -0.047(0.012) | 9.50E-05 |
| | $GP15^n$ | *The percentage of FA2BG2 glycan in total neutral IgG glycans ($GP^n$)* | *$GP15 / GP^n * 100$* | 0.017(0.011) | 1.14E-01 |
| *Neutral IgG glycans - derived parameters* | $G0^n$ | *The percentage of agalactosylated structures in total neutral IgG glycans* | *$SUM(GP1^n: GP6^n)$* | 0.036(0.012) | 3.07E-03 |
| | $G1^n$ | *The percentage of* | *$SUM(GP7^n: GP11^n)$* | -0.026(0.009) | 4.69E-03 |

| | | | | | |
|---|---|---|---|---|---|
| | | *monogalactosylated structures in total neutral IgG glycans* | | | |
| | $G2^n$ | *The percentage of digalactosylated structures in total neutral IgG glycans* | *SUM(GP12ⁿ: GP15ⁿ)* | -0.037(0.012) | 1.72E-03 |
| | $F^{n\ total}$ | *The percentage of all fucosylated (+/- bisecting GlcNAc) structures in total neutral IgG glycans* | *SUM(GP1ⁿ+ GP4ⁿ+ GP5ⁿ+ GP6ⁿ+ GP8ⁿ+ GP9ⁿ+ GP10ⁿ+ GP11ⁿ+ GP14ⁿ+ GP15ⁿ)* | -0.028(0.009) | 1.55E-03 |
| | $FG0^{n\ total}/G0^n$ | *The percentage of fucosylation of agalactosylated structures* | *SUM(GP1ⁿ+ GP4ⁿ+ GP5ⁿ+ GP6ⁿ) / G0ⁿ * 100* | -0.038(0.008) | 6.61E-06 |
| | $FG1^{n\ total}/G1^n$ | *The percentage of fucosylation of monogalactosylated structures* | *SUM(GP8ⁿ+ GP9ⁿ+ GP10ⁿ+ GP11ⁿ) / G1ⁿ * 100* | -0.031(0.009) | 4.04E-04 |
| | $FG2^{n\ total}/G2^n$ | *The percentage of fucosylation of digalactosylated structures* | *SUM(GP14ⁿ+ GP15) / G2ⁿ * 100* | -0.026(0.009) | 3.56E-03 |
| | $F^n$ | *The percentage of fucosylated (without bisecting GlcNAc) structures in total neutral IgG glycans* | *SUM(GP1ⁿ+ GP4ⁿ+ GP5ⁿ+ GP8ⁿ+ GP9ⁿ+ GP14ⁿ)* | -0.084(0.009) | 3.68E-19 |
| | $FG0^n/G0^n$ | *The percentage of fucosylation (without bisecting GlcNAc) of agalactosylated structures* | *SUM(GP1ⁿ+ GP4ⁿ+ GP5ⁿ) / G0ⁿ * 100* | -0.074(0.009) | 2.22E-16 |
| | $FG1^n/G1^n$ | *The percentage of fucosylation (without bisecting GlcNAc) of monogalactosylated structures* | *SUM(GP8ⁿ+ GP9ⁿ) / G1ⁿ * 100* | -0.082(0.009) | 1.60E-18 |

| | | | | | |
|---|---|---|---|---|---|
| | $FG2^n/G2^n$ | *The percentage of fucosylation (without bisecting GlcNAc) of digalactosylated structures* | $GP14^n / G2^n * 100$ | -0.058(0.01) | 1.51E-09 |
| | $FB^n$ | *The percentage of fucosylated (with bisecting GlcNAc) structures in total neutral IgG glycans* | $SUM(GP6^n + GP10^n + GP11^n + GP15^n)$ | 0.09(0.01) | 7.75E-20 |
| | $FBG0^n/G0^n$ | *The percentage of fucosylation (with bisecting GlcNAc) of agalactosylated structures* | $GP6^n / G0^n * 100$ | 0.073(0.009) | 4.22E-15 |
| | $FBG1^n/G1^n$ | *The percentage of fucosylation (with bisecting GlcNAc) of monogalactosylated structures* | $SUM(GP10^n + GP11^n) / G1^n * 100$ | 0.08(0.01) | 5.72E-17 |
| | $FBG2^n/G2^n$ | *The percentage of fucosylation (with bisecting GlcNAc) of digalactosylated structures* | $GP15) / G2^n * 100$ | 0.072(0.01) | 1.90E-12 |
| | $FB^n/F^n$ | *Ratio of fucosylated structures with and without bisecting GlcNAc* | $FB^n / F^n * 100$ | 0.09(0.01) | 3.70E-20 |
| | $FB^n/F^{n\ total}$ | *The incidence of bisecting GlcNAc in all fucosylated structures in total neutral IgG glycans* | $FB^n / F^{n\ total} * 100$ | 0.09(0.01) | 6.23E-20 |
| | $F^n/(B^n + FB^n)$ | *Ratio of fucosylated non-bisecting GlcNAc structures and all structures with bisecting GlcNAc* | $F^n/(GP13^n + FB^n)$ | -0.089(0.01) | 7.74E-20 |
| | $B^n/(F^n + FB^n)$ | *Ratio of structures with bisecting GlcNAc and all fucosylated structures (+/-* | $GP13^n / (F^n + FB^n) * 1000$ | 0.004(0.009) | 6.61E-01 |

| | | | | | |
|---|---|---|---|---|---|
| | | *bisecting GlcNAc)* | | | |
| | $FBG2^n/FG2^n$ | *Ratio of fucosylated digalactosylated structures with and without bisecting GlcNAc* | *$GP15^n/GP14^n$* | 0.074(0.01) | 4.86E-13 |
| | $FBG2^n /(FG2^n + FBG2^n)$ | *The incidence of bisecting GlcNAc in all fucosylated digalactosylated structures in total neutral IgG glycans* | *$GP15^n/(GP14^n + GP15^n) * 100$* | 0.074(0.01) | 5.73E-13 |
| | $FG2^n/(BG2^n + FBG2^n)$ | *Ratio of fucosylated digalactosylated non-bisecting GlcNAc structures and all digalactosylated structures with bisecting GlcNAc* | *$GP14^n/(GP13^n + GP15^n)$* | -0.074(0.01) | 1.23E-12 |
| | $BG2^n/(FG2^n + FBG2^n)$ | *Ratio of digalactosylated structures with bisecting GlcNAc and all fucosylated digalactosylated structures (+/- bisecting GlcNAc)* | *$GP15^n/(GP14^n + GP15^n) * 1000$* | 0.038(0.01) | 2.18E-04 |

*Previously published in Lauc *et al*. 2013

**Online Table II. Descriptive characteristics of the male population included in the validation analysis.**

| Phenotype | TwinsUK<br>Mean(SD) | ORCADES<br>Mean(SD) |
|---|---|---|
| **n** | 189 | 656 |
| **Males %** | 100% | 100% |
| **Age** | 57.21(10.88) | 54.51(14.76) |
| **10-years ASCVD Risk Score** | 11.04(9.29) | 12.19(13.59) |
| **BMI** | 26.70(3.71) | 28.03(5.51) |
| **DBP, mmHG** | 82.03(9.79) | 72.67(9.23) |
| **HDL Cholesterol, mmol/l** | 1.24(0.35) | 1.33(0.37) |
| **SBP, mmHG** | 133.19(16.13) | 132.07(16.26) |
| **Current smokers,%** | 2.65% | 8.52% |
| **Total Cholesterol, mmol/l** | 5.24(1.20) | 5.32(1.12) |
| **T2D, %** | 0% | 4.71% |

**Online Table III. Glycan traits associated with the 10-years ASCVD risk score and their association with smoking, HDL and total cholesterol, systolic blood pressure, type 2 diabetes and insulin resistance adjusting for age, BMI and family relatedness in the discovery cohort.**

| | ASCVD | | SMK | | HDL | | TC | | SBP | | T2D | | HOMA | |
|---|---|---|---|---|---|---|---|---|---|---|---|---|---|---|
| **Glycan** | **Beta (SE)** | **P** | **Beta (SE)** | **P** | **Beta (SE)** | **P** | **Beta (SE)** | **P** | **Beta (SE)** | **P** | **Beta (SE)** | **P** | **Beta (SE)** | **P** |
| **GP6** | 0.07 (0.01) | $1.30x10^{-10}$ | 0.52 (0.08) | $2.08x10^{-10}$ | -0.04 (0.01) | $4.07x10^{-6}$ | 0.07 (0.03) | $1.51x10^{-2}$ | -0.13 (0.34) | $7.01x10^{-1}$ | 0.18 (0.14) | $1.90x10^{-1}$ | 0.06 (0.02) | $9.63x10^{-4}$ |
| **GP14** | -0.06 (0.01) | $1.49x10^{-7}$ | -0.13 (0.1) | $1.80x10^{-1}$ | 0.03 (0.01) | $7.50x10^{-4}$ | -0.15 (0.03) | $9.48x10^{-8}$ | -0.21 (0.36) | $5.56x10^{-1}$ | -0.11 (0.19) | $5.71x10^{-1}$ | -0.03 (0.02) | $1.91x10^{-1}$ |
| **GP18** | -0.08 (0.01) | $5.59x10^{-11}$ | -0.19 (0.09) | $4.71x10^{-2}$ | 0.05 (0.01) | $8.92x10^{-8}$ | -0.15 (0.03) | $2.94x10^{-8}$ | -0.32 (0.35) | $3.55x10^{-1}$ | -0.13 (0.16) | $3.92x10^{-1}$ | -0.05 (0.02) | $4.20x10^{-3}$ |
| **FGS/(F+FG+FGS)** | -0.04 (0.01) | $1.31x10^{-4}$ | -0.01 (0.09) | $8.71x10^{-1}$ | 0.05 (0.01) | $1.27x10^{-8}$ | -0.06 (0.03) | $2.10x10^{-2}$ | -0.35 (0.33) | $2.77x10^{-1}$ | -0.01 (0.14) | $9.40x10^{-1}$ | -0.07 (0.02) | $3.15x10^{-5}$ |
| **FBStotal/FStotal** | 0.05 (0.01) | $4.18x10^{-7}$ | 0.22 (0.09) | $1.03x10^{-2}$ | -0.03 (0.01) | $1.31x10^{-3}$ | 0.08 (0.02) | $8.22x10^{-4}$ | -0.14 (0.31) | $6.46x10^{-1}$ | 0.07 (0.15) | $6.66x10^{-1}$ | 0.01 (0.02) | $7.19x10^{-1}$ |
| **FBS1/FS1** | 0.04 (0.01) | $6.08x10^{-5}$ | 0.12 (0.08) | $1.62x10^{-1}$ | -0.03 (0.01) | $4.25x10^{-4}$ | 0.07 (0.02) | $3.56x10^{-3}$ | -0.1 (0.31) | $7.50x10^{-1}$ | 0.01 (0.15) | $9.38x10^{-1}$ | 0.02 (0.02) | $3.27x10^{-1}$ |
| **FBS1/(FS1+FBS1)** | 0.04 (0.01) | $5.97x10^{-5}$ | 0.11 (0.08) | $1.76x10^{-1}$ | -0.03 (0.01) | $5.63x10^{-4}$ | 0.07 (0.02) | $2.55x10^{-3}$ | -0.11 (0.31) | $7.24x10^{-1}$ | 0.01 (0.16) | $9.31x10^{-1}$ | 0.02 (0.02) | $3.36x10^{-1}$ |
| **GP6n** | 0.1 (0.01) | $7.93x10^{-17}$ | 0.73 (0.09) | $3.33x10^{-15}$ | -0.04 (0.01) | $4.85x10^{-5}$ | 0.13 (0.03) | $5.57x10^{-6}$ | -0.24 (0.35) | $4.99x10^{-1}$ | 0.28 (0.16) | $7.94x10^{-2}$ | 0.04 (0.02) | $3.19x10^{-2}$ |
| **GP9n** | -0.05 (0.01) | $2.58x10^{-7}$ | -0.54 (0.06) | $6.40x10^{-18}$ | 0.02 (0.01) | $1.43x10^{-2}$ | 0.02 (0.02) | $3.25x10^{-1}$ | 0.27 (0.27) | $3.23x10^{-1}$ | -0.18 (0.12) | $1.23x10^{-1}$ | -0.02 (0.01) | $3.02x10^{-1}$ |
| **GP14n** | -0.05 (0.01) | $9.50x10^{-5}$ | -0.01 (0.1) | $9.31x10^{-1}$ | 0.04 (0.01) | $2.03x10^{-5}$ | -0.1 (0.03) | $8.11x10^{-4}$ | -0.29 (0.36) | $4.24x10^{-1}$ | -0.05 (0.16) | $7.76x10^{-1}$ | -0.05 (0.02) | $6.75x10^{-3}$ |
| **GlycA*** | 0.13 (0.01) | $9.34x10^{-21}$ | 0.24 (0.09) | $9.39x10^{-3}$ | -0.09 (0.01) | $3.31x10^{-14}$ | 0.23 (0.03) | $9.70x10^{-12}$ | 1.06 (0.42) | 0.01 | 0.55 (0.17) | $1.30x10^{-3}$ | 0.13 (0.02) | $5.10x10^{-13}$ |

**ASCVD=**10-year atherosclerotic cardiovascular disease risk score adjusting for age, BMI and family relatedness; **SMK**= smoking, **HDL**= HDL cholesterol, **TC**= total cholesterol, **SBP**= systolic blood pressure; **T2D**=type 2 diabetes; **HOMA**=insulin resistance. *GlycA was measured in mmol/L by the NMR metabolomics provider Nightingale inc under the name GP. GlycA has been standardised to have mean 0 and SD 1.

**Online Table IV. Association of glycan traits with the 10-year ASCVD risk score overall and adjusting for smoking, HDL cholesterol, total cholesterol and HOMA respectively in the discovery cohort.**

| | ASCVD | | ASCVD_adj_SMK | | ASCVD_adj_HDL | | ASCVD_adj_TC | | ASCVD_adj_HOMA | |
|---|---|---|---|---|---|---|---|---|---|---|
| **Glycan** | **Beta (SE)** | **P** | **Beta (SE)** | **P** | **Beta (SE)** | **P** | **Beta (SE)** | **P** | **Beta (SE)** | **P** |
| **GP6** | 0.07 (0.01) | $1.30x10^{-10}$ | 0.05 (0.01) | $4.68x10^{-7}$ | 0.05 (0.01) | $4.58x10^{-7}$ | 0.06 (0.01) | $4.54x10^{-9}$ | 0.05 (0.01) | $1.57x10^{-4}$ |
| **GP14** | -0.06 (0.01) | $1.49x10^{-7}$ | -0.06 (0.01) | $6.18x10^{-9}$ | -0.05 (0.01) | $1.39x10^{-5}$ | -0.04 (0.01) | $1.84x10^{-4}$ | -0.05 (0.01) | $9.02x10^{-4}$ |
| **GP18** | -0.08 (0.01) | $5.59x10^{-11}$ | -0.07 (0.01) | $1.86x10^{-11}$ | -0.05 (0.01) | $6.31x10^{-7}$ | -0.06 (0.01) | $4.03x10^{-7}$ | -0.06 (0.01) | $1.24x10^{-5}$ |
| **FGS/(F+FG+FGS)** | -0.04 (0.01) | $1.31x10^{-4}$ | -0.04 (0.01) | $2.96x10^{-5}$ | -0.02 (0.01) | $5.59x10^{-2}$ | -0.03 (0.01) | $1.11x10^{-3}$ | -0.02 (0.01) | $9.62x10^{-2}$ |
| **FBStotal/FStotal** | 0.05 (0.01) | $4.18x10^{-7}$ | 0.05 (0.01) | $1.12x10^{-6}$ | 0.04 (0.01) | $2.55x10^{-5}$ | 0.04 (0.01) | $2.73x10^{-5}$ | 0.05 (0.01) | $2.13x10^{-5}$ |
| **FBS1/FS1** | 0.04 (0.01) | $6.08x10^{-5}$ | 0.04 (0.01) | $1.87x10^{-5}$ | 0.03 (0.01) | $2.72x10^{-3}$ | 0.03 (0.01) | $1.20x10^{-3}$ | 0.04 (0.01) | $8.25x10^{-4}$ |
| **FBS1/(FS1+FBS1)** | 0.04 (0.01) | $5.97x10^{-5}$ | 0.04 (0.01) | $1.56x10^{-5}$ | 0.03 (0.01) | $2.43x10^{-3}$ | 0.03 (0.01) | $1.34x10^{-3}$ | 0.04 (0.01) | $1.01x10^{-3}$ |
| **GP6n** | 0.1 (0.01) | $7.93x10^{-17}$ | 0.07 (0.01) | $5.69x10^{-11}$ | 0.08 (0.01) | $2.90x10^{-13}$ | 0.08 (0.01) | $6.75x10^{-13}$ | 0.08 (0.01) | $7.35x10^{-9}$ |
| **GP9n** | -0.05 (0.01) | $2.58x10^{-7}$ | -0.02 (0.01) | $9.94x10^{-3}$ | -0.04 (0.01) | $6.14x10^{-6}$ | -0.05 (0.01) | $6.92x10^{-9}$ | -0.05 (0.01) | $2.30x10^{-6}$ |
| **GP14n** | -0.05 (0.01) | $9.50x10^{-5}$ | -0.05 (0.01) | $3.02x10^{-6}$ | -0.03 (0.01) | $1.08x10^{-2}$ | -0.03 (0.01) | $3.10x10^{-3}$ | -0.02 (0.01) | $9.85x10^{-2}$ |
| **GlycA** | 0.13 (0.01) | $9.34\ x10^{-21}$ | 0.11 (0.01) | $1.19x10^{-16}$ | 0.09 (0.01) | $2.27x10^{-11}$ | 0.11 (0.01) | $4.35x10^{-15}$ | 0.11 (0.02) | $2.33x10^{-12}$ |

**ASCVD=**10-year atherosclerotic cardiovascular disease risk score adjusting for age, BMI and family relatedness; **ASCVD_adj_SMK**= 10-years ASCVD score adjusting for age, BMI, family relatedness and smoking, **ASCVD_adj_HDL**= 10-years ASCVD scor adjusting for age, BMI, family relatedness and HDL cholesterol, **ASCVD_adj_TC**= 10-years ASCVD scor adjusting for age, BMI, family relatedness and total cholesterol, **ASCVD_adj_HOMA2IR**= 10-years ASCVD scor adjusting for age, BMI, family relatedness and HOMA2IR. Glycan traits in italics do not remain statistically significant after adjustment

for individual risk factors. *****GlycA was measured in mmol/L by the NMR metabolomics provider Nightingale inc under the name GP. GlycA has been standardised to have mean 0 and SD 1.

**Online Table V. Glycan traits associated with the 10-year ASCVD risk score and their association with femoral and carotid plaque in TwinsUK females.**

| | Femoral plaque | | Femoral plaque adj SMK | | Carotid plaque | | Carotid plaque adj SMK | |
|---|---|---|---|---|---|---|---|---|
| **Glycan** | **Beta(SE)** | **P** | **Beta(SE)** | **P** | **Beta(SE)** | **P** | **Beta(SE)** | **P** |
| **GP6** | 0.387(0.152) | **0.01** | 0.359(0.158) | **0.02** | 0.467(0.154) | **$2.40x10^{-3}$** | 0.453(0.157) | **$3.92x10^{-3}$** |
| **GP14** | -0.174(0.165) | 0.29 | -0.177(0.166) | 0.29 | -0.213(0.155) | 0.17 | -0.221(0.156) | 0.16 |
| **GP18** | -0.128(0.162) | 0.43 | -0.144(0.165) | 0.38 | -0.494(0.147) | **$7.70x10^{-4}$** | -0.524(0.152) | **$5.81x10^{-4}$** |
| **FBStotal/FStotal** | 0.021(0.149) | 0.89 | 0.022(0.149) | 0.89 | 0.173(0.133) | 0.19 | 0.177(0.132) | 0.18 |
| **FBS1/FS1** | -0.03(0.148) | 0.84 | -0.003(0.15) | 0.98 | 0.123(0.127) | 0.33 | 0.154(0.13) | 0.23 |
| **FBS1/(FS1+FBS1)** | -0.041(0.149) | 0.78 | -0.014(0.152) | 0.93 | 0.12(0.13) | 0.35 | 0.152(0.133) | 0.25 |
| **GP6n** | 0.48(0.154) | **$1.88x10^{-3}$** | 0.427(0.159) | **0.01** | 0.39(0.152) | **0.01** | 0.347(0.155) | **0.03** |
| **GP9n** | -0.252(0.128) | **0.05** | -0.211(0.129) | 0.10 | -0.297(0.119) | **0.01** | -0.264(0.121) | **0.03** |
| **GlycA*** | 0.317(0.105) | **0.002** | 0.313(0.107) | **0.003** | 0.194(0.099) | **0.05** | 0.298(0.15) | **0.049** |

*GlycA was measured in mmol/L by the NMR metabolomics provider Nightingale inc under the name GP. GlycA has been standardised to have mean 0 and SD 1.

**Online Table VI. Pearson's correlation and p-value between the 8 IgG glycans reproducibly and the NMR GlycA measure in TwinsUK**

| Pearson Correlations | GlycA* | GP6 | GP14 | GP18 | FBStotal/ FStotal | FBS1/FS1 | FBS1/ (FS1+FBS1) | GP6n | GP9n |
|---|---|---|---|---|---|---|---|---|---|
| **GlycA** | 1 | | | | | | | | |
| **GP6** | 0.21<br>$7.7x10^{-18}$ | 1 | | | | | | | |
| **GP14** | -0.22<br>$3.4x10^{-20}$ | -0.69<br>$2.5x10^{-233}$ | 1 | | | | | | |
| **GP18** | -0.21<br>$2.5x10^{-18}$ | -0.74<br>$5.4x10^{-284}$ | 0.87<br>$<10^{-300}$ | 1 | | | | | |
| **FBStotal/FStotal** | 0.17<br>$4.5x10^{-12}$ | 0.50<br>$8.1x10^{-103}$ | -0.73<br>$4.8x10^{-273}$ | -0.76<br>$<10^{-300}$ | 1 | | | | |
| **FBS1/FS1** | 0.16<br>$2.2x10^{-11}$ | 0.50<br>$4.1x10^{-102}$ | -0.70<br>$7.4x10^{-244}$ | -0.77<br>$<10^{-300}$ | 0.97<br>$<10^{-300}$ | 1 | | | |
| **FBS1/(FS1+FBS1)** | 0.16<br>$2.2x10^{-11}$ | 0.50<br>$7.0x10^{-104}$ | -0.70<br>$2.1x10^{-244}$ | -0.77<br>$<10^{-300}$ | 0.97<br>$<10^{-300}$ | 0.99<br>$<10^{-300}$ | 1 | | |
| **GP6n** | 0.20<br>$9.8x10^{-17}$ | 0.94<br>$<10^{-300}$ | -0.78<br>$<10^{-300}$ | -0.76<br>$<10^{-300}$ | 0.55<br>$3.2x10^{-128}$ | 0.51<br>$3.7x10^{-111}$ | 0.52<br>$9.6x10^{-113}$ | 1 | |
| **GP9n** | -0.06<br>$2.4x10^{-2}$ | -0.30<br>$8.0x10^{-36}$ | 0.1369<br>$2.7x10^{-8}$ | 0.111<br>$2.0x10^{-5}$ | -0.10<br>$2.1x10^{-5}$ | -0.08<br>$1.1x10^{-3}$ | -0.08<br>$2.1x10^{-3}$ | -0.34<br>$2.2x10^{-45}$ | 1 |

*GlycA was measured in mmol/L by the NMR metabolomics provider Nightingale inc under the name GP. GlycA has been standardised to have mean 0 and SD 1.

**Online Table VII. Association between GP18 and various measures of cholesterol, lipoproteins and triglycerides in serum from TwinsUK females**

| Lipid trait | Beta | SE | P |
|---|---|---|---|
| **Apolipoprotein A-I** | 0.069 | 0.055 | $2.09x10^{-1}$ |
| **Apolipoprotein B** | -0.148 | 0.052 | $4.86x10^{-3}$ |
| **Concentration of chylomicrons and extremely large VLDL particles** | -0.210 | 0.053 | $9.73x10^{-5}$ |
| **Concentration of IDL particles** | -0.070 | 0.052 | $1.80x10^{-1}$ |
| **Concentration of large HDL particles** | 0.149 | 0.054 | $5.70x10^{-3}$ |
| **Concentration of large LDL particles** | -0.083 | 0.053 | $1.19x10^{-1}$ |
| **Concentration of large VLDL particles** | -0.226 | 0.053 | $2.29x10^{-5}$ |
| **Concentration of medium HDL particles** | 0.093 | 0.050 | $6.36x10^{-2}$ |
| **Concentration of medium LDL particles** | -0.082 | 0.054 | $1.30x10^{-1}$ |
| **Concentration of medium VLDL particles** | -0.228 | 0.052 | $1.56x10^{-5}$ |
| **Concentration of small LDL particles** | -0.089 | 0.053 | $9.23x10^{-2}$ |
| **Concentration of small VLDL particles** | -0.213 | 0.050 | $2.25x10^{-5}$ |
| **Concentration of very large HDL particles** | 0.095 | 0.055 | $8.66x10^{-2}$ |
| **Concentration of very large VLDL particles** | -0.224 | 0.054 | $3.49x10^{-5}$ |
| **Concentration of very small VLDL particles** | -0.149 | 0.049 | $2.64x10^{-3}$ |
| **Remnant cholesterol (non-HDL, non-LDL -cholesterol)** | -0.138 | 0.049 | $5.16x10^{-3}$ |
| **Sphingomyelins** | -0.045 | 0.058 | $4.41x10^{-1}$ |
| **Triglycerides in chylomicrons and extremely large VLDL** | -0.209 | 0.053 | $9.24x10^{-5}$ |
| **Triglycerides in HDL** | -0.079 | 0.045 | $8.26x10^{-2}$ |
| **Triglycerides in IDL** | -0.086 | 0.046 | $6.48x10^{-2}$ |
| **Triglycerides in large HDL** | 0.092 | 0.047 | $4.93x10^{-2}$ |
| **Triglycerides in large LDL** | -0.060 | 0.047 | $2.07x10^{-1}$ |
| **Triglycerides in large VLDL** | -0.223 | 0.052 | $1.85x10^{-5}$ |
| **Triglycerides in LDL** | -0.063 | 0.049 | $1.97x10^{-1}$ |
| **Triglycerides in medium HDL** | -0.093 | 0.048 | $5.38x10^{-2}$ |
| **Triglycerides in medium LDL** | -0.038 | 0.050 | $4.51x10^{-1}$ |
| **Triglycerides in medium VLDL** | -0.227 | 0.051 | $1.31x10^{-5}$ |
| **Triglycerides in small LDL** | -0.106 | 0.048 | $2.94x10^{-2}$ |

| | | | |
|---|---|---|---|
| **Triglycerides in small VLDL** | -0.213 | 0.050 | $2.81x10^{-5}$ |
| **Triglycerides in very large HDL** | -0.048 | 0.050 | $3.37x10^{-1}$ |
| **Triglycerides in very large VLDL** | -0.223 | 0.053 | $2.71x10^{-5}$ |
| **Triglycerides in very small VLDL** | -0.165 | 0.048 | $6.04x10^{-4}$ |
| **Triglycerides in VLDL** | -0.197 | 0.048 | $5.10x10^{-5}$ |

**Online Figure I.** Glycan traits significantly associated with 10-years ASCVD risk in females and validation in males.

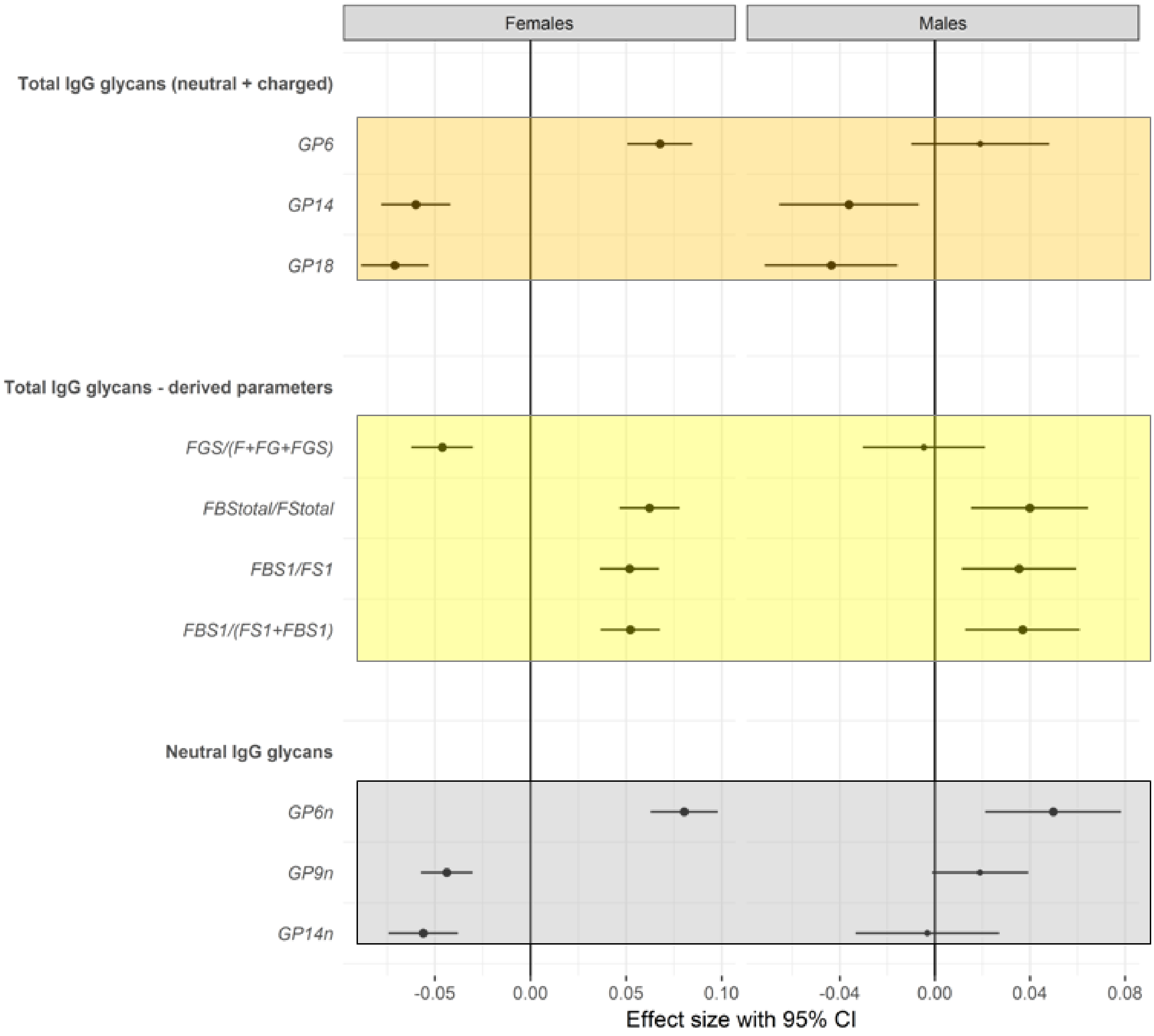

Supplement: Supplementary file 1 [file res-122-1555-s001.pdf]
